# Comparative Transcriptomic and Metabolic Analyses Reveal the Molecular Mechanism of Ovule Development in the Orchid, *Cymbidium sinense*

**DOI:** 10.3389/fpls.2021.814275

**Published:** 2022-01-21

**Authors:** Danqi Zeng, Caixia Que, Jaime A. Teixeira da Silva, Shutao Xu, Dongmei Li

**Affiliations:** ^1^Key Laboratory of South China Agricultural Plant Molecular Analysis and Genetic Improvement, Provincial Key Laboratory of Applied Botany, South China Botanical Garden, Chinese Academy of Sciences, Guangzhou, China; ^2^Guangdong Provincial Research Center for Standardization of Production Engineering Technology of Orchids, Shunde Polytechnic, Foshan, China; ^3^College of Life Sciences, University of the Chinese Academy of Sciences, Beijing, China; ^4^Independent Researcher, Kagawa, Japan; ^5^College of Innovative Design, City University of Macau, Taipa, Macao SAR, China

**Keywords:** ovule development, differentially expressed unigenes, plant hormones, *Cymbidium sinense*, metabolomics

## Abstract

Ovule development is pivotal to plant reproduction and seed development. *Cymbidium sinense* (Orchidaceae) has high ornamental value due to its pleasant aroma and elegant floral morphology. The regulatory mechanism underlying ovule development in orchids, especially *C. sinense*, is largely unknown and information on the *C. sinense* genome is very scarce. In this study, a combined analysis was performed on the transcriptome and non-targeted metabolomes of 18 *C. sinense* ‘Qi Jian Hei Mo’ ovule samples. Transcriptome analysis assembled gene-related information related to six growth stages of *C. sinense* ovules (S1-S6, equivalent to 30, 35, 42, 46, 53, and 60 days after pollination). Illumina sequencing technology was used to obtain the complete set of transcriptome sequences of the 18 samples. A total of 81,585 unigene sequences were obtained after assembly, 24,860 (30.47%) of which were functionally annotated. Using transcriptome sequencing technology, a total of 9845 differentially expressed unigenes (DEUs) were identified in *C. sinense* ovules that were assigned to specific metabolic pathways according to the Kyoto Encyclopedia of Genes and Genomes (KEGG). DEUs associated with transcription factors (TFs) and phytohormones were identified and analyzed. The TFs homeobox and MADS-box were associated with *C. sinense* ovule development. In particular, the phytohormones associated with DEUs such as indole-3-acetic acid (IAA), cytokinin (CK), gibberellin (GA), abscisic acid (ABA), brassinosteroid (BR), and jasmonate (JA), may have important regulatory effects on *C. sinense* ovule development. Metabolomic analysis showed an inconsistent number of KEGG annotations of differential metabolites across comparisons (S2_vs_S4, S2_vs_S5, and S4_vs_S5 contained 23, 26, and 3 annotations, respectively) in *C. sinense* ovules. This study provides a valuable foundation for further understanding the regulation of orchid ovule development and formation, and establishes a theoretical background for future practical applications during orchid cultivation.

## Introduction

*Cymbidium sinense* (Orchidaceae) is an economically important flowering orchid with high ornamental value, elegant color, and delicate scent (Huang and Dai, 1998; Kim et al., 2016). Members of the Orchidaceae not only account for a high proportion of all flowering plants, they also have highly diversified and specialized flower morphology. Extensive research has been conducted on the molecular regulation and genetic regulation of floral development in orchids (Xu et al., 2006; Aceto and Gaudio, 2011; Zhang et al., 2013; Teixeira da Silva et al., 2014). The Orchidaceae has a unique flower type with a characteristic column (Rudall and Bateman, 2002). Pollinia are found at the top of the column while the ovary, which develops post-pollination, is found at the bottom of it (Yu and Goh, 2001). The ovule develops into a seed after fertilization and ensures normal reproduction and the production of offspring (Schneitz, 1999), which is regarded as an important biological process during plant development.

Although the ovule in animals is sometimes considered as the ovum or egg cell, the ovule of angiosperms (seed plants) can have a variety of roles throughout development given its complex structure, including integument(s), the micropyle and nucellus (Rudall, 2021). In Arabidopsis (*Arabidopsis thaliana*), ovules are initiated from the carpels during flower development (Smyth et al., 1990). There are also numerous reports on the regulation of ovule growth and development in Arabidopsis (Gross-Hardt et al., 2002; Sieber et al., 2004; Colombo et al., 2008; Liu et al., 2019), such as the INO transcription factor (TF), which regulates the distal-proximal mode of the ovule (Villanueva et al., 1999). Ovule development is also very complex in rice (*Oryza sativa*): research focused on the morphology and cellular characteristics of ovules (Lopez-Dee et al., 1999; Locato and De Gara, 2018) and on the regulatory mechanism of development, noting that plant hormones play a vital role in embryonic development (Bencivenga et al., 2011). Separately, sequencing revealed the developmental process of rice ovules at the gene expression level (Kubo et al., 2013; Wu et al., 2015, 2017). In the Bromeliaceae, ovule-specific features have been described in detail (Fagundes and de Araujo Mariath, 2014; Mendes et al., 2014), as well as embryological characteristics of the ovule (Sajo et al., 2004), ovule morphology (Kuhn et al., 2016), and other aspects related to the ovule (Novikoff and Odintsova, 2008; Nogueira et al., 2015).

Some research on ovule development has been conducted in the Orchidaceae. Early studies identified genes expressed predominantly in the ovules of *Phalaenopsis* (Nadeau et al., 1996). The functional genes related to ovule development in *Phalaenopsis* and *Dendrobium* have been characterized, with C- and D-class MADS-box genes, such as *PeMADS1* and *DOAG2*, respectively, playing an important role in ovule development in these two orchid genera (Chen et al., 2012; Wang et al., 2020). Other studies found an increasingly important role of plant hormones in ovule development in the Orchidaceae (Nadeau et al., 1993; Ketsa and Rugkong, 2000; Tsai et al., 2008), in particular, critical regulatory role of ethylene in ovary maturation and differentiation. Compared with other orchids, very little research has been conducted on the regulation of ovule development in *Cymbidium*. Only a few ovule-related genes have been cloned from *Cymbidium*, especially *C. sinense*, with some reports focusing on the morphology and development of the *C. sinense* ovule (Swamy, 1942; Yeung et al., 1994; Yeung and Law, 1997). The molecular mechanisms in *C. sinense* ovules is still poorly understood.

Transcriptomic data can be used to glean a global understanding of gene expression in individual organisms at a certain point in time or in a specific tissue (Denoeud et al., 2008; Alagna et al., 2009; Dassanayake et al., 2009). Some studies on orchids have reported the key role of cDNA sequencing in discovering functional candidate genes in floral development, flowering time, and other developmental events, for example in *Phalaenopsis* and *Dendrobium* (Hsiao et al., 2006; Xu et al., 2006), as well as *Oncidium* and *Vanda* (Tan et al., 2005; Teh et al., 2011). Despite these studies, the molecular mechanism underlying ovule development in the Orchidaceae has received limited attention. In particular, for *C. sinense*, a comprehensive description of the complete complement of expressed genes is still unavailable, and candidate genes related to ovule development are poorly understood (Zhang et al., 2013). A critical in-depth study of the *C. sinensis* ovule is required to unravel its structural and developmental aspects as well as the underlying mechanism as a way to further *Cymbidium* breeding programs and develop a cultivation mechanism with high-yielding cultivars.

In this study, we performed an integrated transcriptomic and metabolomic analysis to investigate global changes in gene expression and metabolites during the development of *C*. *sinense* ovules. Our results shed light on the molecular mechanism of ovule development in this orchid.

## Materials and Methods

### Plant Materials and Experimental Design

The flowers of *C. sinense* ‘Qi Jian Hei Mo,’ which was used in this study, were manually pollinated. Plants were grown and maintained in pots in a greenhouse at the Jiu Wan Orchid Field, in Foshan, China. The greenhouse was well ventilated, relative air humidity was 75–80%, day/night temperatures were 28/25°C with a 12-h photoperiod. Six developmental stages (S1–S6) of *C*. *sinense* ovules were collected from 12 fruits, frozen rapidly in liquid nitrogen and kept at –80°C until RNA extraction. Biological triplicates for each stage were used for RNA sequence (RNA-seq) analysis. Ovules at S2, S4, and S5 were collected to analyze metabolites, with six biological replicates for each stage.

### RNA Extraction

Total RNA of each sample was extracted using the Quick RNA extraction kit (Huayueyang Biotechnology Co. Ltd., Beijing, China). Tissues were ground to a fine powder using a mortar and pestle after freezing them in liquid nitrogen. Powder was transferred into an RNase-free centrifuge tube, which contained RNA extraction buffer, and vortexed rapidly for 30 s, performed according to the manufacturer’s protocol. The integrity and content of total RNA was determined by a NanoDrop 2000c Spectrophotometer (Thermo Fisher Scientific, Wilmington, NC, United States) and an Agilent 2100 Bioanalyzer (Agilent Technologies Inc., Palo Alto, CA, United States). Three microgram of total RNA of each sample with an RNA integrity number (RIN) value ≥7.5 and concentration ≥ 100 ng/μL were used for cDNA library preparation and sequencing by Biomarker Technologies Inc. (Beijing, China).

### cDNA Library Preparation and Sequencing

mRNA enrichment from total RNA was performer using oligo d(T)_25_ magnetic beads (New England Biolabs Inc., Ipswich, MA, United States). mRNA was used for library preparation with the NEBNext^®^ Ultra™ RNA Library Prep Kit (New England Biolabs Inc.). The insert size of libraries was estimated by the Agilent 2100 bioanalyzer, and the effective concentration was assayed by quantitative real-time (RT-qPCR). The libraries were sequencing using an Illumina Novaseq 6000 Sequencing System to produce raw reads. To obtain clean reads from raw reads, reads containing an adapter or ploy-N, as well as the poor-quality reads, were filtered out. These analyses were completed by Biomarker Technologies Inc.

### *De novo* Assembly and Functional Annotation

The total clean reads generated by sequencing the 18 libraries were used for *de novo* full-length transcriptome reconstruction using Trinity (Grabherr et al., 2013) to generate transcripts. The assembled transcripts were clustered by Corset (Davidson and Oshlack, 2014) to remove redundant transcripts. Remaining transcripts were defined as unigenes. The integrity of assembled transcripts was evaluated with Benchmarking Universal Single-Copy Orthologs (BUSCO^1^) (Waterhouse et al., 2018).

Using BLASTx alignment (*E*-value ≤ 10^––5^), the unigenes previously obtained were annotated with seven functional annotation databases, including the National Center for Biotechnology non-redundant protein sequences database (nr^2^), Clusters of Orthologous Groups of proteins (COG^3^), a manually annotated and reviewed protein sequence database (Swissprot^4^), Protein family (Pfam^5^), Kyoto Encyclopedia of Genes and Genomes (KEGG^6^), evolutionary genealogy of genes: Non-supervised Orthologous Groups (eggNOG^7^) and Gene Ontology (GO^8^). GetOrf (EMBOSS v. 6.3.1) used to predict the amino acid sequences of unigenes. Sequences were aligned in Pfam by HMMER software suite (v. 3.0) (E-value ≤ 10^–10^). These analyses were completed by the authors in collaboration with Biomarker Technologies Inc.

### Identification of Transcription Factor

To identify the TFs in *C. sinense* ovules, a program to identify and classify plant TFs, iTAK (Zheng et al., 2016),^9^ and a prediction tool provided by a plant TF database (Tian et al., 2020),^10^ were used to identify and classify the TFs. The predicted TFs from the two methods were combined as the total TFs in *C. sinense*.

### Identification of Differentially Expressed Unigenes

Based on the *de novo* assembled data, the clean reads produced from each sample were mapped to the reference unigenes with RSEM (Li and Dewey, 2011) to obtain read counts of each sample. Read counts were then converted to fragments per kilobase of transcript sequence per millions (FPKM) to quantify transcript abundance. For differential expression analysis, read counts were used for normalization, estimate fold-change and calculate false discovery rate (FDR) using the DESeq method in the DESeq2 package (Love et al., 2014). A unigene with an absolute value of log_2_ (fold change) > 1 and an FDR < 0.05 between the six stages was defined as a differentially expressed unigene (DEU). These analyses were completed by the authors in collaboration with Biomarker Technologies Inc.

### Metabolic Profiling

To screen out metabolites with important biological significance and with significant changes during the development of *C. sinense* ovules, three biological replicates of S1–S6 were analyzed, giving a total of 18 samples. We used a mixer mill (MM 400, Retsch, Haan, Germany) to homogenize the three freeze-dried biological samples of each stage, by adding zirconia beads (Biospec, BioSpec Products Inc., Bartlesville, OK, United States), and applying 30 Hz for 90 s. Samples of 20 ± 1 mg were transferred into 2 mL EP tubes (Axygen Scientific Inc., Silicon Valley, CA, United States), 500 μL of precooled extract (methanol (CNW Technologies, Duesseldorf, Germany; 67-56-1, HPLC): chloroform (Adamas, Basel, Switzerland; 67-66-3, HPLC = 3:1, v/v) was added, then 10 μL of adonitol (Sigma-Aldrich, Burlingtonm MA, United States; 488-81-3, ≥99%) was added. Samples were homogenized for 30 s using a vortex oscillator (Vortex-Genie 2, Scientific Industries, New York, NY, United States). Samples were immediately ground by a grinding mill (JXFSTPRP-24, Jingxin Industrial Development Co. Ltd., Shanghai, China) for 4 min at 35 Hz. The extraction mixture was treated in an ultrasonic apparatus (YM-080S, Fangao Microelectronics Co. Ltd, Shenzhen, China) for 5 min at 40°C while incubated in ice water, then centrifuged at 4°C for 15 min, at 10,000 rpm. The supernatant (100 μL) was carefully collected and transferred to a fresh 1.5 mL EP tube. A 30 μL aliquot of supernatant from each sample was mixed as the quality control (QC) sample. The supernatant was collected and completely dried in a vacuum concentrator (Huamei Biochemical Instrument Factory, Taicang, China), to which 60 μL of methoxyamination hydrochloride (20 mg/mL in pyridine) was added and incubated for 30 min at 80°C. Samples were immediately derivatized with 60 μL of *bis*-(trimethylsilyl)-trifluoroacetamide (including 1% trimethylchlorosilane, v/v) reagent (REGIS Technologies, Chicago, IL, United States) and incubated for 90 min at 70°C. Lastly, 5 μL of fatty acid methyl esters (dissolved in chloroform) reagent (Dr. Ehrenstorfer GmbH, Augsburg, Germany) was added to the QC sample, and progressively cooled in the laboratory to 37°C. Samples were further analyzed by GC-TOF-MS, which was conducted using an Agilent 7890 gas chromatography system (Agilent, Atlanta, GA, United States) coupled with a Pegasus HT time-of-flight mass spectrometer (LECO Corp., St. Joseph, MI, United States). The system utilized a DB-5MS capillary column (30 m × 250 μm inner diameter, 0.25 μm film thickness; J&W Scientific, Folsom, CA, United States). The settings are described next. A 1 μL aliquot of sample was used to run the analysis in splitless mode. Helium was used as the carrier gas, the purge flow of the front inlet was 3 mL min^–1^, and the gas flow rate across the column was 1 mL min^–1^. The initial temperature was kept at 50°C for 60 s, before increasing to 310°C at a rate of 10°C min^–1^, then kept for 8 min at 310°C. The ion source temperatures, transfer line, and injection were 250, 280, and 280°C, respectively. The energy was –70 eV in the electron impact mode. After a solvent delay of 6.35 min, the mass spectrometry data were acquired in full-scan mode with the *m/z* range of 50–500 at a rate of 12.5 spectra per second. The metabolites of 18 *C*. *sinense* ovule samples were delivered to, and quantitatively analyzed by, Biomarker Technologies Inc.

### Identification of Differential Metabolites

The identification of differentially accumulated metabolites (DAMs) was based on an online METLIN batch metabolite search (Progenesis QI^11^) and Biomark’s self-built library^12^ for metabolite identification. Theoretical fragment identification was simultaneously carried out. The mass number deviations were all within 100 per mil (‰). Details such as data filtering, peak detection, comparison, calculation, and identification of DAMs are described in Tang et al. (2019). Among them, metabolites with FC > 2, *t*-test *P*-value < 0.05 and variable importance in projection (VIP) > 1 were considered as DAMs, which were mapped to the KEGG metabolic pathway for pathway enrichment analysis (FDR ≤ 0.05) (Kanehisa et al., 2008), including the use of the KEGG database,^13^ the human metabolome database (HMDB^14^) and clusterProfiler R package^15^ to annotate the identified metabolites. These analyses were completed by the authors in collaboration with Biomarker Technologies Inc.

## Results

### Sequencing and *de novo* Assembly of *C. sinense* Transcriptome and Functional Annotation

To study the system-wide changes in the *C. sinense* ovule during differential developmental stages (S1–S6), we obtained the transcriptomic data of 18 *C. sinense* ovule samples. The statistical results of transcriptome sequencing are shown in Table 1. After removing low-quality reads and all possible contaminations, we obtained a total of 115.15 Gb of clean data; each sample has at least 6.14 Gb of clean data (Table 1). For each sample, more than 94.58% of bases had a score of ≥Q30 and the GC content of clean reads was similar (between 45.52 and 46.69%). This indicates that the obtained data are high quality data, and sequencing results could be used for subsequent analyses.

**TABLE 1:** Sequencing statistics of *Cymbidium sinense* transcriptome during ovary development.

Sample	Number of clean reads	GC content (%)	% ≥ Q30 (%)	Mapped reads (%)	Mapped ratio
S1-1	21,187,529	45.81	95.73	18,189,961	85.85
S1-2	20,558,304	45.95	95.71	17,700,640	86.10
S1-3	20,467,384	46.01	95.65	17,629,023	86.13
S2-1	20,755,996	46.13	95.37	17,763,370	85.58
S2-2	20,235,253	46.12	95.33	17,379,672	85.89
S2-3	22,035,045	45.97	95.48	19,016,783	86.30
S3-1	22,962,814	46.69	95.09	19,984,499	87.03
S3-2	21,612,735	46.40	94.78	18,416,292	85.21
S3-3	21,190,300	46.53	94.94	18,062,959	85.24
S4-1	20,929,960	45.82	95.20	18,070,071	86.34
S4-2	20,900,762	46.14	95.45	18,154,223	86.86
S4-3	19,595,266	45.72	95.07	16,814,367	85.81
S5-1	22,375,253	45.68	95.23	19,202,416	85.82
S5-2	20,440,396	45.90	94.58	17,540,777	85.81
S5-3	23,577,643	45.89	95.16	20,235,517	85.83
S6-1	22,860,183	45.52	95.11	19,766,873	86.47
S6-2	20,797,301	45.97	95.40	17,849,805	85.83
S6-3	21,865,298	46.25	95.22	18,968,763	86.75

*S1-1, S1-2, and S1-3 indicate the three biological replicates of stage 1 [30 days after pollination (DAP)]. S2-1, S2-2, and S2-3 indicate the three biological replicates of stage 2 (35 DAP). S3-1, S3-2, and S3-3 indicate the three biological replicates of stage 3 (42 DAP). S4-1, S4-2, and S4-3 indicate the three biological replicates of stage 4 (46 DAP). S5-1, S5-2, and S5-3 indicate the three biological replicates of stage 5 (53 DAP). S6-1, S6-2, and S6-3 indicate the three biological replicates of stage 6 (60 DAP).*

**FIGURE 1:**
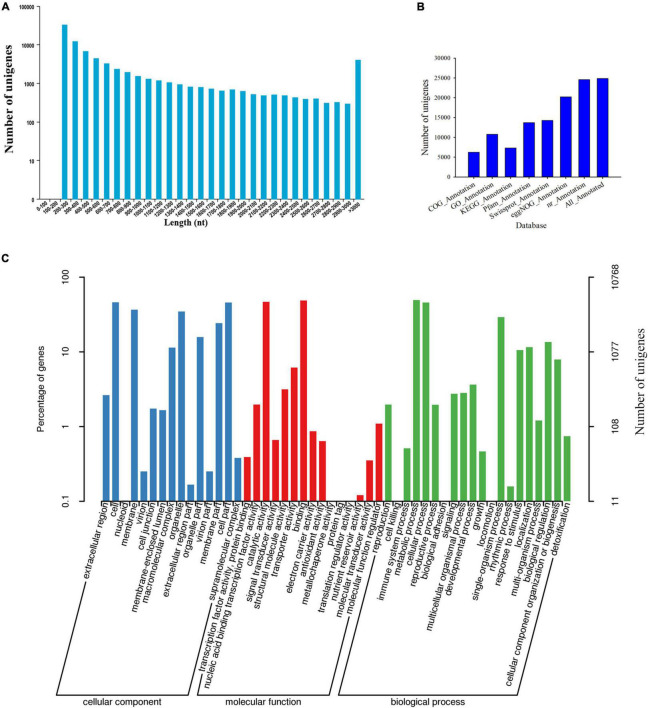
Information of *de novo* assembled unigenes in *C*. *sinense* gleaned from transcriptomic analysis. **(A)** Length distribution of *de novo* assembled unigenes. **(B)** Annotation of unigenes against seven public databases (COG, GO, KEGG, Pfam, Swissprot, eggNOG, and nr). **(C)** GO functional classification (three subcategories) of the unigenes. The left Y-axis refers to the percentage of a specific category of target genes in that main category, while the right Y-axis refers to the number of target genes expressed in each subcategory.

First, the clean reads generated by sequencing of the transcriptome were aligned and assembled into contigs using Trinity software. Contigs were clustered and partially assembled to obtain transcripts, and then the most important transcript was selected as the unigene sequence. After further clustering and assembly, we obtained 81,585 unigenes in total (Supplementary Table 1). The N50 of the unigenes is 1,605, and 16,826 unigenes were longer than 1,000 bp (Figure 1A and Supplementary Table 1). As shown in Figure 1A, the specific distribution of unigenes indicates that the sequence assembly of the *C. sinense* ovule is relatively complete.

Second, the 24,860 unigenes found in the *C. sinense* ovule were functionally annotated using seven public databases (COG, GO, KEGG, Pfam, SwissProt, eggnog, and nr) (Figure 1B). Of all unigenes, 30.47% were similar to known gene sequences in all databases (Supplementary Table 2).

To evaluate the functional role of target genes, their functional categories were classified according to their GO enrichment. A total of 10,768 genes (Supplementary Table 2) were assigned to at least one GO term, accounting for 43.31% of the functional categories of all 24,860 unigenes that were successfully annotated. These GO terms were categorized into 50 functional groups which were divided into three categories, including biological processes, cellular components and molecular functions (Figure 1C), which might play important roles during ovule development. In the cellular component category, the unigenes were chiefly related to “cell,” “cell part,” “membrane,” and “organelle.” The categories “binding” and “catalytic activity” were the predominant terms among the GO molecular function category. Within the biological processes category, “metabolic process,” “cellular process,” and “single-organism process” were the most abundant terms. In particular, the highest percentage of “metabolic process” among the biological processes suggests that a large number of new genes might be involved in secondary metabolite synthesis in *C. sinense* ovules.

### Identification of Transcription Factors in *C. sinense*

**FIGURE 2:**
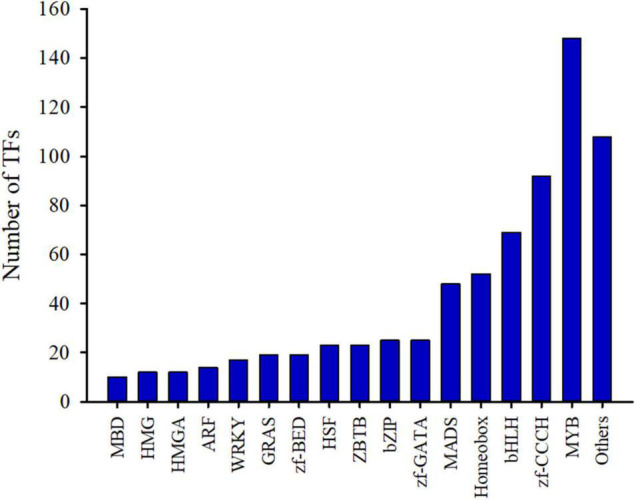
Identification of transcription factors (TFs) from *C*. *sinense* ovules.

TFs can recognize specific DNA sequences and regulate the expression of target genes, and are key regulators for regulating various biological processes (Mitsis et al., 2020). In order to promote functional gene research in *C. sinense*, a transcriptome-wide identification of TFs in *C. sinense* was performed. The MYB TF family accounted for the largest proportion (20.67%) of these genes, followed by 148 members, as well as zf-CCCH and bHLH with 92 and 69 members, respectively (Figure 2 and Supplementary Table 3). Of particular note, two TF families, homeobox and MADS, which play various roles in plant growth and development, also accounted for a relatively large proportion, containing 52 and 48 members, respectively (Supplementary Table 3). These growth-related TFs might be involved in the development of *C. sinense* ovules.

### Identification and Clustering Analysis of Differentially Expressed Unigenes During *C. sinense* Ovule Development

**FIGURE 3:**
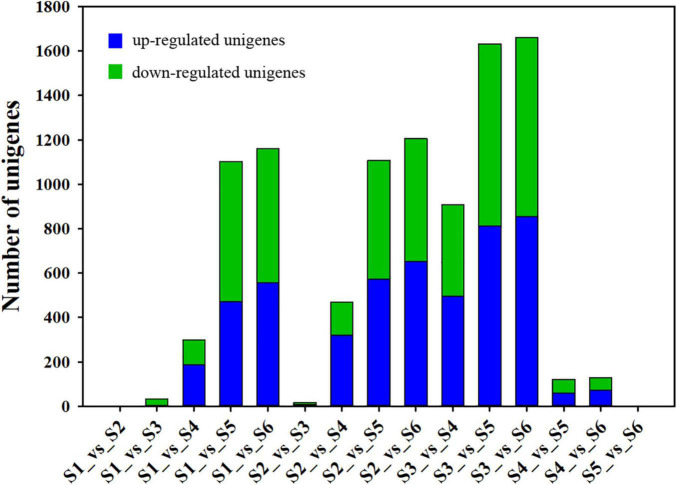
Identification of differential expressed unigenes by pairwise comparisons. “S1_vs_S2” represents “the comparison between S1 and S2” or “S1 compared to S2”. The same applies to all other comparisons between S1 and S6.

To clarify the key DEUs that regulate ovule development, a threshold with a log_2_ ratio >1 and *P* < 0.05 were used in pairwise comparisons to identify DEUs. Gene expression analysis showed that the expression of 9,845 unigenes was differentially altered in paired comparison of the six growth stages of *C. sinense* ovules (Supplementary Table 4). As shown in Figure 3, the pairwise comparison of DEUs during ovule growth and development (except for S1_vs_S2 and S5_vs_S6 comparisons) indicated that a total of 5,065 unigenes were up-regulated while 4,780 unigenes were down-regulated. Among them, S3_vs_S6 had the largest number of DEUs, followed by S3_vs_S5, S2_vs_S6 and S1_vs_S6 comparisons, while no DEUs were detected in S1_vs_S2 and S5_vs_S6 comparisons. This implies that the S3 period of *C. sinense* ovule development might be a key node of drastic changes in gene expression, and that the periods when unigenes displayed obvious differential expression lay between S3 and S5 or S6.

**FIGURE 4:**
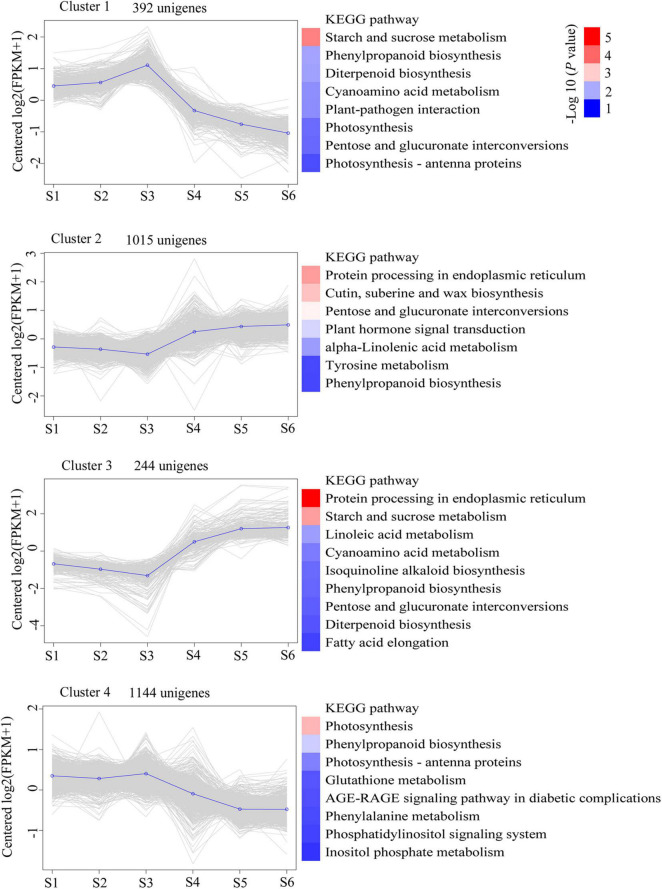
Clustering analysis of time-course RNA-seq revealed transcriptional changes during development of the *C*. *sinense* ovule. Blue lines highlight the centroid of each cluster. The KEGG pathway categories with a *P*-value < 0.05 are shown. The color codes represent the -log10 (*P* value).

To determine significant changes in KEGG pathways, we used DEU co-expression analysis. Furthermore, we performed *k*-means cluster analysis on 9,845 DEUs to decipher the overall trend of gene expression profiles. The clustering analysis indicated that key transcriptional activation or repression occurred during *C*. *sinense* ovule development. The 9,845 DEUs were grouped into four clusters during *C*. *sinense* ovule development (Figure 4). In cluster 1, S3 was the peak value (highest point), and the most enriched KEGG pathway is “starch and sucrose metabolism”. This suggests that changes to genes in this pathway might be due to energy demands of the developing ovule. Clusters 2 and 3 displayed similar expression trends: S3 was the lowest point; overall, changes were relatively flat in S1–S3 and S4–S6. The most enriched KEGG pathway was “protein processing in endoplasmic reticulum”. This suggests that protein processing was active during later stages of ovule development (S4–S6). In cluster 4, like cluster 1, the peak value was observed in S3 with the most enriched KEGG pathway being “photosynthesis”. These results indicate that S3 is a key point of transcriptional changes, in which several key functional unigenes involved in “starch and sucrose metabolism,” “protein processing in endoplasmic reticulum,” “photosynthesis,” and “phenylpropanoid biosynthesis” were highly enriched from among the six growth and development stages of the *C. sinense* ovule.

### Characterization of Functional Genes Involved in Starch and Sucrose Metabolism, and Phenylpropanoid Biosynthesis

To obtain a more comprehensive understanding of transcript expression in *C. sinense* ovules at different developmental stages, we identified DEUs involved in starch and sucrose metabolism and phenylpropanoid biosynthesis during S1–S6 (Tables 2, 3).

**TABLE 2:** Homologous sequences of DEUs involved in starch and sucrose metabolism during six growth and developmental stages of *C*. *sinense* ovules.

Gene ID	Description	S1	S2	S3	S4	S5	S6
c61950.graph_c0	Beta-fructofuranosidase	1.25	0.52	0.57	1.92	1.65	1.68
c63373.graph_c0	Fructokinase	4.06	3.04	7.34	2.22	1.21	1.03
c59725.graph_c0	Beta-glucosidase	1.82	1.09	1.08	6.81	11.85	6.36
c63927.graph_c0	Beta-glucosidase	1.05	0.89	0.13	3.87	12.19	8.44
c60343.graph_c0	Endoglucanase	31.98	17.7	44.66	13.96	14.05	12.79
c62140.graph_c0	Endoglucanase	0	0.03	0	0.07	2.01	3.17
c61840.graph_c0	Trehalase	24.92	28.28	26.87	17.94	12.99	11.02
c62609.graph_c1	Trehalose-phosphate phosphatase	7.17	6.85	9.27	5.86	3.19	2.68
c59506.graph_c0	Trehalose-phosphate phosphatase	7.58	6.38	4.35	15.82	34.67	31.37
c62634.graph_c0	Trehalose-phosphate phosphatase	106.15	133.1	120.87	75.28	35.64	34.49
c48295.graph_c0	Beta-amylase	4	5.11	5.6	2.3	1.49	1.43
c34186.graph_c0	Beta-amylase	8.08	9.95	13.56	3.55	5.17	6.69

*The number in each stage indicates the average FPKM. More details about S1–S6 can be found in the materials and methods.*

**TABLE 3:** Homologous sequences of DEUs involved in phenylpropanoid biosynthesis during six growth and developmental stages of *C. sinense* ovules.

Gene ID	Description	S1	S2	S3	S4	S5	S6
c32911.graph_c0	Shikimate *O*-hydroxycinnamoyltransferase	0.15	0.17	0	0.66	1.06	2.66
c63253.graph_c0	Caffeic acid 3-*O*-methyltransferase	6.67	4.57	1.27	16.6	22.9	21.98
c66522.graph_c5	Caffeic acid 3-*O*-methyltransferase	21.96	27.36	29.73	17	17.43	14.13
c57919.graph_c0	Cinnamyl alcohol dehydrogenase	24.7	27.86	43.26	20.69	12.93	14.84
c60400.graph_c0	Peroxidase	1.62	1.71	2.37	0.8	1.16	0.53
c65092.graph_c0	Peroxidase	112.83	84.05	100.01	224.89	156.8	186.38
c56929.graph_c0	Peroxidase	3.53	3.27	5.94	3.88	1.38	1.56
c36841.graph_c0	Peroxidase	4.24	4.24	2.89	11.58	9.14	11.07
c36064.graph_c0	Peroxidase	2.19	1.32	1.97	0.06	0.18	0.08
c36619.graph_c0	Peroxidase	1.67	0.67	1.35	4.77	1.62	1.89
c56253.graph_c0	Peroxidase	123.79	124.29	111.99	117.35	196.35	208.12
c67154.graph_c0	Peroxidase	67.96	53.94	76.33	33.58	22.13	21

*The number in each stage indicates average of FPKM. More details about S1–S6 can be found in the materials and methods.*

Starch and sucrose are important compounds in the growth and development of plants. Based on bioinformatics analysis of the *C*. *sinense* transcriptome, DEUs assigned to the “starch and sucrose metabolism” metabolic pathway were highly represented. Two unigenes (c59725.graph_c0 and c63927.graph_c0), encoding beta (β)-glucosidase, were up-regulated in S4–S6, with the highest expression in S4 (Table 2). The up-regulation of these two β-glucosidase unigenes at a late development stage suggests that related substances catalyzed by β-glucosidase increased in this stage of ovule development. The expression of an endoglucanase (c60343.graph_c0) unigene peaked in S3, but the overall expression of another endoglucanase unigene (c62140.graph_c0) was up-regulated during ovule development. Endoglucanase and β-glucosidase act synergistically to hydrolyze cellulose (Singhania et al., 2013). These results suggest that the hydrolysis of cellulose might occur during the development of the *C. sinense* ovule. Two trehalose 6-phosphate phosphatase unigenes (c59506.graph_c0 and c61840.graph_c0) were differentially expressed during ovule development. The expression of one trehalose 6-phosphate phosphatase unigene (c61840.graph_c0) unigene in S1, S2, and S3, peaking in S3, was significantly higher than in S4, S5, and S6, dipping in S6, while the expression of another (c59506.graph_c0) unigene peaked in S5, and its overall expression was higher in the later stages of growth and development of the *C. sinense* ovule (i.e., S4–S6) than in the early growth period (S1–S3). This indicates that these two trehalose 6-phosphate phosphatase unigenes may play different roles in *C*. *sinense* ovule development. Overall, the expression of two β-amylase (c48295.graph_c0 and c34186.graph_c0) unigenes peaked in S3. β-Amylase mediates maltose production from starch breakdown (Fulton et al., 2008), suggesting that stored starch was hydrolyzed and used as nutrients by the growing ovule. In addition, more than half of the expression of genes was highest during S3, indicating that this period might be critical for the “starch and sucrose metabolism” metabolic pathway.

The phenylpropanoid metabolic pathway is one of the main pathways for plants to synthesize secondary metabolic compounds. Further analysis revealed that 12 DEUs were identified as homologous sequences of the major genes related to the “phenylpropanoid biosynthesis” metabolic pathway, among which four types of DEUs were annotated (Table 3). Cinnamyl alcohol dehydrogenase (CAD) catalyzes the last step in the biosynthesis of monolignols, which are lignin precursors (Baucher et al., 1996). CAD expression level was high in S1–S2, but peaked in S3, suggesting that lignin might accumulate during ovule development. It is worth noting that eight peroxidase (POD) unigenes were discovered among the DEUs assigned to the “phenylpropanoid biosynthesis” pathway, and the expression levels of four POD unigenes (c60400.graph_c0, c56929.graph_c0, c36064.graph_c0, and c36619.graph_c0) were low while those of another two POD unigenes (c65092. graph_c0 and c56253.graph_c0) were very high, ranging from 84.05 to 224.89 FPKM. Of the remaining two PODs, one (c36841.graph_c0) had a higher expression level in the late period of germinal development i.e., S4–S6, while the other (c67154.graph_c0) had a higher expression level in the early period of growth and development i.e., S1–S3. The POD family of enzymes is involved in lignin biosynthesis (Yang et al., 2007; Herrero et al., 2013). Taken together, we speculate that lignin accumulated during ovule development.

### Differential Metabolite Screening

**FIGURE 5:**
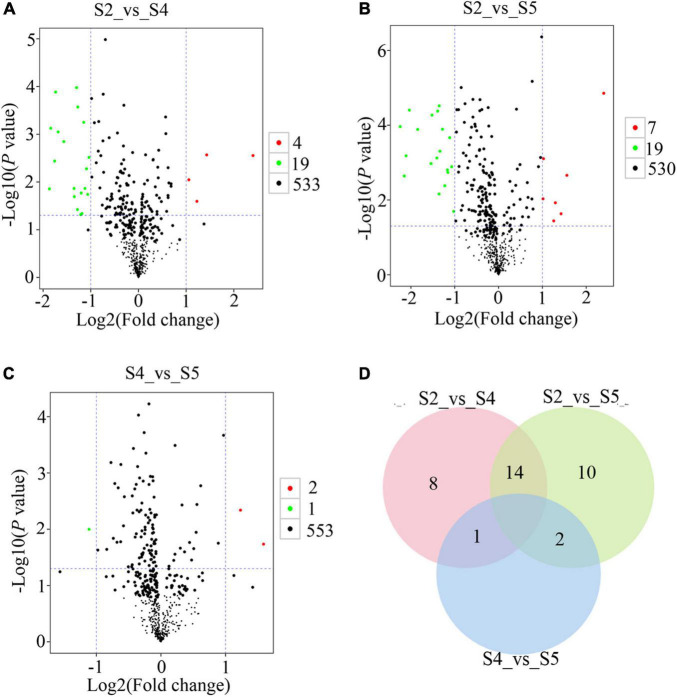
Metabolomic GC-TOF-MS-based analyses of the *C*. *sinense* ovule at three developmental stages (S2, S4 and S5). Visualization, using volcano plots, of the identified metabolites from S2_vs_S4 **(A)**, S2_vs_S5 **(B)**, and S4_vs_S5 **(C)** comparisons. Green dots in the graph represent down-regulated DAMs, red dots represent up-regulated DAMs, and black represents metabolites that were detected but were not significantly different. **(D)** Venn diagram for the number of unique and overlapping DAMs from S2_vs_S4, S2_vs_S5 and S4_vs_S5 comparisons.

**FIGURE 6:**
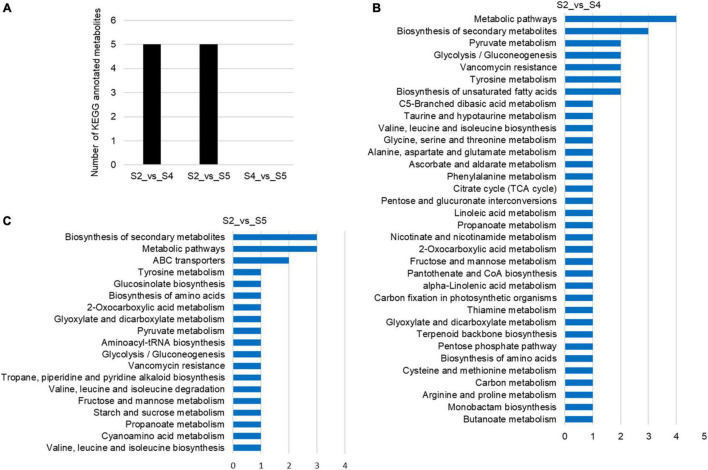
KEGG annotations of differentially accumulated metabolites (DAMs) of the *C*. *sinense* ovule at three developmental stages (S2, S4, and S5). **(A)** Number of KEGG-annotated metabolites. Visualization, using a bar graph, of the identified metabolites from S2_vs_S4 **(B)**, and S2_vs_S5 **(C)**.

To explore metabolic networks during *C. sinense* ovule development, an untargeted metabolomics method i.e., gas chromatography coupled with time-of-flight mass spectrometry (GC-TOF-MS), was used to detect the metabolites in S1–S6. In total, 18 samples were analyzed, revealing 556 metabolites. The identified metabolites were demonstrated using volcano plots and Venn diagrams. Generally speaking, gene expression is spatio-temporal (Larochelle et al., 1999; Kudapa et al., 2018), so changes in substances are expected to lag behind gene expression. Jointly observing Figures 3, 4, it can be seen that S3 is a key node of changes in gene expression, so S4, the stage following S3, was selected for the study of metabolites. In addition, a comparison between S1 and S2 (S1_vs_S2) and S5_vs_S6 gene expression changed slowly and no DEUs were detected, so S2 and S5 were randomly selected for metabolomic analysis. Consequently, in order to gain insight into the changes to metabolites among developmental stages, DAMs between groups were identified when VIP was ≥1, fold-change was ≥2 or ≤0.5, and *P* value was <0.05. Here, three periods (S2, S4, and S5) were compared to obtained a volcano map for three pairwise comparisons (S2_vs_S4, S2_vs_S5, and S4_vs_S5), to visualize the identified metabolites. Moreover, we identified a total of 52 DAMs (23 in S2_vs_S4, 26 in S2_vs_S5, and three in S4_vs_S5 comparisons). Among the identified DAMs, the S2_vs_S4 comparison showed that four were up-regulated and 19 were down-regulated (Figure 5A and Supplementary Table 5), seven were up-regulated and 19 were down-regulated in the S2_vs_S5 comparison (Figure 5B and Supplementary Table 5), and very few metabolites showed significant differences, with only two being up-regulated and one down-regulated in the S4_vs_S5 comparison (Figure 5C and Supplementary Table 5). Furthermore, in order to compare and analyze the relationships between DAMs between groups, a Venn diagram was drawn based on the number of DAMs obtained from each of the three groups. The results showed that there were overlapping metabolites between groups (Figure 5D and Supplementary Tables 6–8), such as “analyte 80,” “lactic acid,” “linoleic acid methyl ester,” “galactinol 2,” etc. Among these DAMs, 14 were the same in the S2_vs_S4 and S2_vs_S5 comparison (including up- and down-regulation), one was the same in the S2_vs_S4 and S4_vs_S5 comparison, and two were the same in the S2_vs_S5 and S4_vs_S5 comparisons. Additionally, eight and 10 unique DAMs were found in S2_vs_S4 and S2_vs_S5 comparisons, respectively. According to the KEGG annotation, five DAMs in the S2_vs_S4 comparison were assigned to 34 KEGG pathways, with four DAMs in “metabolic pathways” and three DAMs in “biosynthesis of secondary metabolites” (Figures 6A,B). In the S2_vs_S5 comparison, both “metabolic pathways” and “biosynthesis of secondary metabolites” contained three DAMs (Figures 6A,C). However, no DAMs assigned to any KEGG pathway were found in the S4_vs_S5 comparison (Figure 6A). These results suggested that the biosynthesis of metabolites was inactive during *C*. *sinense* ovule development.

### Differential Expression Analysis of Homeobox and MADS-Box Unigenes at Different Developmental Stages

**FIGURE 7:**
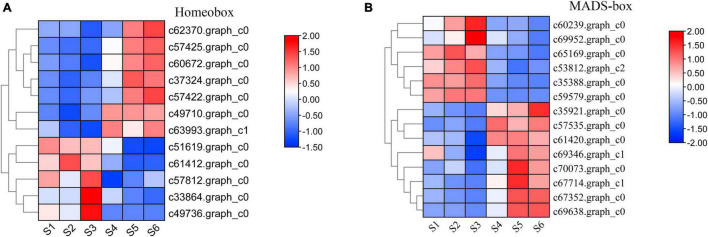
Expression pattern of development-related genes during six growth stages of the *C*. *sinense* ovule. Expression patterns of DEUs annotated from the homeobox **(A)** and MADS-box **(B)** superfamilies. The color of each box represents log2(FPKM + 1) values, negative values (blue) represent down-regulation and positive values (red) represent up-regulation. Only DEUs from these two superfamilies are shown.

A large number of studies found that the TFs of homeobox and MADS-box genes are closely related to plant growth and development (Ramachandran et al., 1994; Jack, 2001; Holland, 2013). Based on the results of TFs identified in *C. sinense* (Figure 2), 52 (7.26%) homeobox and 48 (6.70%) MADS-box unigenes were identified (Supplementary Table 3). We selected homeobox and MADS-box unigenes for further expression pattern analysis by plotting the FPKM values of unigenes that were differentially expressed at S1-S6 in the *C. sinense* ovule to draw a heat map (Figure 7).

A total of 12 homeobox DEUs were identified, and their expression patterns were roughly divided into two clusters (Figure 7A). The expression level of seven DEUs (c62370.graph_c0, c57425.graph_c0, c60672.graph_c0, c37324.graph_c0, c57422.graph_c0, c49710.graph_c0, and c63993.graph_c1) in the upper part of the heat map was low in S1-S3, high in S4-S6, and peaked in S6 (five DEUs) or S5 (two DEUs). In contrast, the expression patterns of five DEUs (c51619.graph_c0, c61412.graph_c0, c57812.graph_c0, c33864. graph_c0, and c49736.graph_c0) in the lower part of the heat map were opposite to those of the aforementioned seven DEUs: their expression level in S1–S3 was higher than in S4–S6, peaking in S3 (3 DEUs) or S1 (2 DEUs). It should be emphasized that one DEU (c62370.graph_c0) had the highest overall expression level, the lowest expression at S3, and highest expression at S6. Homeobox genes thus appear to play a role in the growth and development of the *C. sinense* ovule.

Similarly, 14 MADS-box DEUs were identified and their expression patterns were also roughly divided into two clusters (Figure 7B). The expression levels of six DEUs (c60239.graph_c0, c69952.graph_c0, c65169.graph_c0, c53812.graph_c2, c35388.graph_c0, and c59579.graph_c0) in the upper part of the heat map, S1–S3 were higher than in S4–S6, except for one DEU (c65169.graph_c0) which showed highest expression in S2, while the remaining five DEUs showed highest expression in S3. The remaining eight DEUs (C35921.graph_c0, c57535.graph_c0, c61420.graph_c0, c69346.graph_c1, c70073.graph_c0, c67714.graph_c1, c67352.graph_c0, and c69638.graph_c0) in the lower part of the heat map showed an opposite trend to the upper part, and their overall trends are essentially opposite to those of homeobox DEUs. Moreover, six DEUs displayed highest expression in S5. In particular, the overall expression level of one MADS-box DEU (c69638.graph_c0) was much higher than other DEUs, especially in S5 and S6, suggesting that this DEU might play an important role in the growth and development of the *C. sinense* ovule.

### Functional Categorization of Deferentially Expressed Genes

**FIGURE 8:**
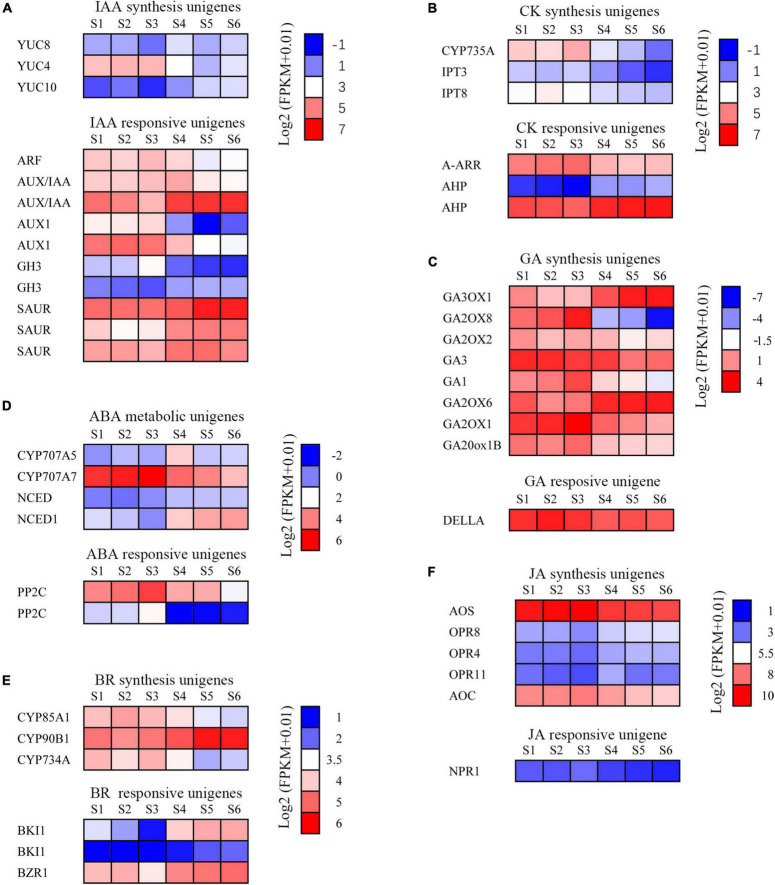
Changes of plant hormone synthesis and signal transduction pathway unigenes during the development of the *C*. *sinense* ovule. **(A)** Expression analysis of auxin synthesis signal transduction pathway unigenes. ARF, auxin response factor; AUX/IAA, auxin/indole acetic acid protein; GH3, Gretchen Hagen; SAUR, small auxin-up RNAs; YUC, indole-3-pyruvate monooxygenase. **(B)** Expression analysis of cytokinin synthesis and signal transduction pathway unigenes. A-ARR, type-A Arabidopsis response regulator; AHP, histidine-containing phosphotransfer protein; CYP735A, cytokinin hydroxylase; IPT, adenylate isopentenyltransferase. **(C)** Expression analysis of GA synthesis and signal transduction pathway unigenes. GA, Ent-kaurene oxidase; GA2OX, Gibberellin 2-beta-dioxygenase; GA20ox1B, Gibberellin 20 oxidase 1-B; GA3OX, Gibberellin 3-beta-dioxygenase. **(D)** Expression analysis of ABA synthesis and signal transduction pathway unigenes. CYP707A5, CP450; abscisic acid 8’-hydroxylase; NCED, 9-*cis*-epoxycarotenoid dioxygenase; PP2C, protein phosphatase-2C. **(E)** Expression analysis of brassinosteroid synthesis and signal transduction pathway unigenes. BKI1, BRI1 kinase inhibitor 1-like; BZR1, protein BZR1 homolog 1-like; CYP734A, cytochrome P450 734A; CYP85A1, cytochrome P450 85A1; CYP90B1, cytochrome P450 90B1. **(F)** Expression analysis of JA synthesis and signal transduction pathway genes. AOC, Allene oxide cyclase; AOS, Allene oxide synthase; NPR1, BTB/POZ domain and ankyrin repeat-containing protein NPR1-like; OPR, 12-oxophytodienoate reductase. The color of each box represents the log_2_(FPKM + 1) value.

To understand the changes in transcript pattern of unigenes involved in plant hormone synthesis and the signal transduction pathway during six stages of development of the *C. sinense* ovule, heat map analysis was used to analyze the relationships between them. Our results indicate that there were six major categories of plant hormones identified, namely IAA (Figure 8A), CK (Figure 8B), GA (Figure 8C), abscisic acid (ABA; Figure 8D), BR (Figure 8E), and JA (Figure 8F). Each category was divided into synthesis unigenes and responsive unigenes. These hormones are closely related to plant vegetative and reproductive growth, fruit ripening senescence, seed dormancy, and other functions.

The IAA synthesis-related unigenes included YUC8, YUC4, and YUC10 (Figure 8A). The overall expression level of YUC8 and YUC10 was low. The expression level of YUC4 was high during early stages of growth and development (S1–S3), but showed a decreasing trend in later stages (S4–S6), suggesting that this gene might play a role in immature tissues of *C. sinense* ovules. In addition, five types of unigenes responded to IAA: ARF, AUX/IAA (two homologous unigenes), AUX1 (two unigenes), CH3 (two unigenes), and SAUR (three unigenes). The expression of SAUR increased whereas that of ARF and AUX1 decreased as the ovule grew and developed. The expression patterns of the two homologous unigenes of AUX/IAA and GH3 were inverse during ovule growth and development. These differences in the expression of IAA-responsive unigenes suggest a complex IAA-related response during *C. sinense* ovule development.

The expression of three CK synthesis-related unigenes all peaked in S2 or S3, then decreased in later stages of development (S4–S6, Figure 8B). The expression of A-ARR, a CK-responsive unigene, showed a downward trend with ovule development while AHP (two unigenes) showed an upward trend (Figure 8B). In addition, there were eight GA synthetic unigenes, including of *GA3*, whose expression level decreased during ovule growth and development (Figure 8C), suggesting that it might play a similar or co-regulatory role with the IAA synthesis unigene YUC4 at an early stage of *C. sinense* ovule development. The only GA-responsive unigene, DELLA, had a high overall expression level and few differences in S1–S6 (Figure 8C). Moreover, CYP707A7, a ABA synthesis-related unigene, had a high expression level, peaking at S3, and showed a downward trend in S4–S6 (Figure 8D). Furthermore, the expression of BR synthesis unigenes CYP85A1 and CYP734A showed a decreasing trend in S4–S6, while CYP90B1 showed an opposite trend. The expression levels of BR-responsive unigenes were slightly different, while the expression trends of the two BKI1 were inconsistent (Figure 8E). Finally, from among the five JA synthesis-related unigenes, two (AOS and AOC) were highly expressed whereas the expression levels of the remaining three (OPR8, OPR4, and OPR11) were almost indistinguishable among S1–S6 (Figure 8F). In summary, their expression profiles were either up- or down-regulated at different developmental stages, indicating that these unigenes had an important role in the regulation of *C. sinense* ovule development. In particular, several key unigenes such as YUC4, GA3, and CYP707A7, played a special role in regulating the development of the *C*. *sinense* ovule. These results suggest that plant hormones played a role in *C. sinense* ovule development.

## Discussion

### Transcriptional Sequencing and *de novo* Assembly of a Non-reference Genome Orchid, *C. sinense*

Ovules are the female sexual organ of higher plants that develop into seeds after fertilization (Shi and Yang, 2011). Ovule development, which is one of the most vital events in high plants because offspring are produced through sexual reproduction, plays a crucial role in plant evolution and the cultivation of new varieties (Yeung et al., 1994; Nadeau et al., 1996; Schneitz, 1999). *C*. *sinense* is a popular ornamental and economically important flower (potted and cut flowers) in the world and its seedlings are obtained by tissue culture (Xu et al., 2006; Aceto and Gaudio, 2011). However, new varieties of *C*. *sinense* with novel appearances and quality are annually in demand.

Transcriptional sequencing is an important method to obtain gene sequences and identify key genes involved in developmental events and metabolic pathways. To the best of our knowledge, there are no comprehensive studies on ovule development in *C*. *sinense*. Notably, *C*. *sinense* can only produce a massive number of ovules in each ovary after successful pollination (Yeung and Law, 1997), and the ovules of *C*. *sinense* develop and mature about 45 days after pollination, and can generally be divided into six stages. Consequently, to shed light on this process, transcriptional sequencing and *de novo* assembly of unigenes were performed, while changes in metabolites during ovule development were also estimated. In total, 81,585 unigenes were *de novo* assembled with an average length of 1857.33 bp (Figure 1A and Supplementary Table 1). In the orchid, *Dendrobium officinale*, 93,881 unigenes with a mean length of 790 bp were obtained using transcriptional sequencing (Zhang et al., 2016). In *Phalaenopsis*, 26,565 unigenes was annotated using transcriptional sequencing (Xu et al., 2015). KEGG pathway annotation showed that DEUs were mainly enriched in several pathways, such as “starch and sucrose metabolism,” “phenylpropanoid biosynthesis,” “protein processing in endoplasmic reticulum,” “photosynthesis,” and “plant hormone signal transduction” (Figure 4), suggesting that these pathways, particularly the first two, might be associated with differences among S1–S6 stages in ovule development. In the “phenylpropanoid biosynthesis” pathway, shikimate *O*-hydroxycinnamoyltransferase is involved in the biosynthesis of phenylpropanoid amides (PAs) (Kang et al., 2009). PAs are related to plant growth and development such as flower formation (Facchini et al., 2002). The caffeic acid 3-*O*-methyltransferase (OMT) family members *AtCCOMT2* and *AtCCOMT4-7* were expressed in the inflorescence stems of *Arabidopsis* (Raes et al., 2003). CAD is closely related to fruit development and floral organs (Kim et al., 2007; Singh et al., 2010). These results imply that PAs, OMT, and CAD may be indirectly involved in the regulation of ovule development. Unfortunately, these DEUs have not been reported in other plants, including orchids, in relation to ovule development. Therefore, this not only provides a good basis for discovering the expression of genes related to ovule development in *C*. *sinense*, but also indicates that transcriptional sequencing is a powerful tool for obtaining novel gene sequences.

### Transcription Factors Are Involved in Plant Ovule Development

TFs are involved in developmental progression by regulating the expression of related target genes (Spitz and Furlong, 2012). Development of the ovule, a complex structure and precursor of a seed, requires the participation of a diversity of TFs. TFs related to ovule formation have been identified in *Jatropha curcas* L. (Xu et al., 2019) and *Arabidopsis* (Kelley and Gasser, 2009). Homeobox superfamily TFs are widely involved in plant development, including ovule development. A homeodomain TF, BELL1 (BEL1), is required for altering of both auxin distribution and patterning of the *Arabidopsis* ovule through exogenous cytokinin treatment (Bencivenga et al., 2012). The homeobox superfamily TF WUS gene, belonging to the WOX family, is specifically expressed in ovules and is responsible for ovule development in a range of plants (van der Graaff et al., 2009). Another *WOX* gene *PRETTY FEW SEEDS2* regulates ovule development in *Arabidopsis* (Park et al., 2005). In this study, 52 homeobox superfamily TFs were identified as DEUs (Figure 2 and Supplementary Table 3). These might play a role in orchid ovule development. MADS-box family TFs are well known for their regulation of flower patterning, and increasing studies are revealing that MADS-box TFs are also involved in ovule development. For example, SEEDSTICK (STK; formerly known as AGL11), SHATTERPROOF1 (SHP1; formerly known as AGL1), and SHATTERPROOF 2 (SHP2; formerly known as AGL5) are responsible for ovule development, since *agl11 shp1 shp2* triple mutants displayed a phenotype in which ovules were converted into carpel-like or leaf-like structures in *Arabidopsis* (Pinyopich et al., 2003). The B_sister_ MADS-box gene was found during ovule development in petunia (*Petunia hybrida*) and *Arabidopsis*, and was critical for determining the identity of the endothelial layer within the ovule (de Folter et al., 2006). In our study, 48 DEUs belonging to the MADS-box family were identified (Figure 2 and Supplementary Table 3), suggesting that MADS-box TFs might act as regulators in *C. sinense* ovule development.

### Phytohormones May Play a Role in *C. sinense* Ovule Development

Phytohormones are fundamental players in the initiation of ovule formation. In cotton (*Gossypium hirsutum*), the content of endogenous phytohormones such as IAA, CK, ABA, and GA, as well as enzyme activities related with IAA and cytokinin metabolism, changed as the ovule developed (Zhang et al., 2009). In *Arabidopsis*, auxin (IAA) accumulated in ovules after fertilization and acts as an important role in early embryo patterning (Robert et al., 2018). In our study, three indole-3-pyruvate monooxygenase genes were differentially expressed during ovule development in *C. sinense* (Figure 8). The genes involved in CK, GA, and ABA biosynthesis and their signal transduction pathway also changed during six different ovule development stages (Figure 8). These results indicate that IAA, CK, ABA and GA may play a role in the development of the *C. sinense* ovule. Furthermore, BR can be perceived by membrane-localized kinase, namely BRASSINOSTEROID-INSENSITIVE 1 (Hothorn et al., 2011; She et al., 2011). BR is responsible for ovule integument growth in *A. thaliana* (Jia et al., 2020). Exogenous application of a BR, brassinolide, promoted fiber elongation, while a BR biosynthesis inhibitor repressed fiber development of *in vitro*-cultured cotton ovules (Sun et al., 2005). Our data showed that three genes were involved in BR-biosynthesis and three acted as BR-responsive genes whose expression was altered during ovule development in *C. sinense* (Figure 8E). This suggests that BR might play a role in the development of the ovule of this orchid.

## Conclusion

In this study, we employed transcriptomic and metabolomic analyses to investigate the molecular and metabolic mechanisms controlling ovule development in *C. sinense*. We identified growth- and development-related TFs such as homeobox and MADS-box TFs, as well as plant hormones such as auxin, CK and BR, which may be feasibly involved in *C. sinense* ovule development. These TFs and phytohormones, as important regulators of ovule development, might play various and specific roles during *C. sinense* ovule development, providing comprehensive information to reveal the expression of relevant genes or various biological pathways of ovule development in the Orchidaceae. In addition, DAMs were identified in this paper by pairwise comparisons at six different developmental stages, although they are not directly reported to be associated with ovule development. Therefore, we speculate that the biosynthesis of metabolites was inactive during *C. sinense* ovule development. Overall, our results provide important clues that reveal the molecular mechanism underlying *C. sinense* ovule development, and aim to indicate some significant yet unexplored dynamics. In conclusion, the present results reveal that further research on the molecular mechanism underlying *C. sinense* ovule development can start from two pathways, the first being TFs related to ovule development identified in this study, and the other being hormone metabolism. Understanding the mechanisms of ovule development in orchids could lead toward orchid improvement, and will ultimately diversify the orchid market. Further genetic and biochemical analyses would help understand the regulatory mechanism of orchid development.

## Data Availability Statement

The original contributions presented in the study are publicly available. This data can be found here: National Center for Biotechnology Information (NCBI) BioProject database under accession number PRJNA783745.

## Author Contributions

DL: conceptualization, resources, supervision, and project administration. DZ and DL: methodology. DZ and SX: software and validation. DZ and JT: formal analysis, writing—review and editing, and visualization. DZ: investigation. DZ and CQ: data curation and writing—original draft preparation. All authors have read and agreed to the published version of the manuscript.

## Conflict of Interest

The authors declare that the research was conducted in the absence of any commercial or financial relationships that could be construed as a potential conflict of interest.

## Publisher’s Note

All claims expressed in this article are solely those of the authors and do not necessarily represent those of their affiliated organizations, or those of the publisher, the editors and the reviewers. Any product that may be evaluated in this article, or claim that may be made by its manufacturer, is not guaranteed or endorsed by the publisher.

## Footnotes

1
http://busco.ezlab.org/

2
http://www.ncbi.nlm.nih.gov

3
http://www.ncbi.nlm.nih.gov/COG

4
https://www.expasy.org/

5
http://pfam.xfam.org/

6
https://www.kegg.jp/

7
http://eggnog.embl.de/

8
http://geneontology.org/

9
http://bioinfo.bti.cornell.edu/tool/itak

10
http://planttfdb.gao-lab.org/

11
http://non-linear.com/

12
http://www.biocloud.net/

13
http://www.genome.jp/kegg/

14
http://www.hmdb.ca/

15
http://www.bioconductor.org/
