# Supplementary material for: Comparative Transcriptomic and Metabolic Analyses Reveal the Molecular Mechanism of Ovule Development in the Orchid, Cymbidium sinense
Source: Front Plant Sci. 2022 Jan 21;12:814275. doi: 10.3389/fpls.2021.814275 (PMC8813969; doi:10.3389/fpls.2021.814275)
Supplement: Supplementary file 1 [file Data_Sheet_1.PDF]

Supplementary Material

1 Supplementary Tables

Table S1. Transcriptome sequencing data assembly results statistics table.

Table S2. Statistics of unigenes annotated in public databases.

Table S3. Statistics of the number of annotated transcription factors.

Table S4. Statistics of all differentially expressed genes (DEGs).

Table S5. Statistics of differentially expressed metabolites (DEMs) at three developmental stages (S2, S4, and S5).

Table S6. Differentially expressed metabolites (DEMs) from the S2_vs_S4 comparison.

Table S7. Differentially expressed metabolites (DEMs) from the S2_vs_S5 comparison.

Table S8. Differentially expressed metabolites (DEMs) from the S4_vs_S5 comparison.

1.1 Supplementary Tables

Table S1. Transcriptome sequencing data assembly results statistics table.

Length range (bp)	Transcripts Number (relative %)	Unigenes Number (relative %)
200-300	39,109 (13.31%)	32,335 (39.63%)
300-500	29,718 (10.11%)	18,940 (23.22%)
500-1000	41,410 (14.09%)	13,484 (16.53%)
1000-2000	71,551 (24.34%)	8,694 (10.66%)
2000+	1 12,146 (38.15%)	8,132 (9.97%)
Total number	293,934	81,585
Total length (bp)	545,933,475	64,357,560
N50 length (bp)	2,881	1,605
Mean length (bp)	1857.33	788.84

Notes: “Length range” indicates the different length intervals of unigenes; numbers in the table indicate the number of unigenes in the corresponding length range while the percentage values in parentheses indicate the relative proportion of unigenes in the corresponding length range; “Total number” indicates the total number of assembled unigenes; “Total length” indicates the total length of assembled unigenes; “N50 length” indicates the largest length such that at least 50% of all base pairs (bp) are contained in contigs of this length or larger; “Mean length” indicates the average length of unigenes.

Table S2. Statistics of unigenes annotated in public databases.

# Annotated database	Annotated number	Length (≥ 300 and <1000, bp)	Length (≥ 1000, bp)
COG_Annotation	6,260	933	4,755
GO_Annotation	10,768	2,574	6,702
KEGG_Annotation	7,312	1,574	5,011
KOG_Annotation	12,369	2,903	7,895
Pfam_Annotation	13,711	2,832	9,902
Swissprot_Annotation	14,267	3,339	9,550
eggNOG_Annotation	20,258	5,230	12,431
nr_Annotation	24,573	7,217	13,237
All_Annotated	24,860	7,272	13,254

Notes: “Annotated databases” denotes each functional database that was examined; “Annotated number” denotes the number of unigenes annotated to each database; “Length (≥ 300 and <1000)” denotes the number of unigenes whose length is greater than or equal to 300 and less than 1000 base airs (bp) annotated to each database; “Length (≥ 1000)” denotes the number of unigenes with a length greater than 1000 bp annotated to each database.

Table S3. Statistics of the number of annotated transcription factors.

# TF name	Annotated number
MBD	10
HMG	12
HMGA	12
ARF	14
WRKY	17
GRAS	19
zf-BED	19
HSF	23
ZBTB	23
bZIP	25
zf-GATA	25
MADS	48
Homeobox	52
bHLH	69
zf-CCCH	92
MYB	148
Others	108
MBD	10
HMG	12

Note: TF, transcription factor

Table S4. Statistics of all differentially expressed genes (DEGs).

DEG comparison	All DEGs	Up-regulated	Down-regulated
S1_vs_S3	34	0	34
S1_vs_S4	299	186	113
S1_vs_S5	1102	471	631
S1_vs_S6	1161	556	605
S2_vs_S3	16	8	8
S2_vs_S4	469	321	148
S2_vs_S5	1107	573	534
S2_vs_S6	1206	652	554
S3_vs_S4	909	496	413
S3_vs_S5	1631	813	818
S3_vs_S6	1659	856	803
S4_vs_S5	122	60	62
S4_vs_S6	130	73	57
Total	9845	5065	4780

Notes: “DEG comparison” indicates the name of sets of differentially expressed genes that were compared among the six developmental stages; “All DEGs” indicates the number of DEGs in each comparisons; “Up-regulated” indicates the number of up-regulated DEGs; “Down-regulated” indicates the number of down-regulated DEGs.

Table S5. Statistics of differentially expressed metabolites (DEMs) at three developmental stages (S2, S4, and S5).

Comparison	Number of DEMs	Down-regulated DEMs	Up-regulated DEMs
S2_vs_S4	23	19	4
S2_vs_S5	26	19	7
S4_vs_S5	3	1	2

Table S6. Differentially expressed metabolites (DEMs) from the S2_vs_S4 comparison.

#ID	DEM name	Fold change	*P* value	VIP	Regulation
meta_54	Analyte 80	0.337958	0.001426	1.806919	down
meta_56	Pyruvic acid	0.434979	0.01704	1.504267	down
meta_62	lactic acid	5.287343	0.002796	1.88842	up
meta_159	2-ketoadipate 4	2.079668	0.008988	1.765898	up
meta_248	Halostachine 1	0.299751	0.000131	1.996586	down
meta_262	Analyte 371	0.414156	0.000268	1.90024	down
meta_307	4-Acetamidobutyric acid 1	0.311236	0.000893	1.828745	down
meta_347	4-Hydroxyphenylethanol	0.279416	0.000748	1.872498	down
meta_398	unknown	0.40674	0.000106	1.939236	down
meta_403	Synephrine 2	0.459653	0.013631	1.458302	down
meta_480	glucose 1	2.696914	0.002707	1.816005	up
meta_516	unknown	0.411909	0.037822	1.292224	down
meta_538	Linoleic acid methyl ester	0.392381	0.013668	1.549984	down
meta_561	linoleic acid	0.393841	0.020262	1.466088	down
meta_563	linolenic acid	0.440228	0.04585	1.316502	down
meta_573	pyridoxal phosphate 1	0.487256	0.003052	1.731322	down
meta_604	Analyte 847	0.27416	0.013934	1.571855	down
meta_606	D-erythro-sphingosine 2	2.339647	0.025405	1.398382	up
meta_661	Monoolein	0.43179	0.048246	1.280894	down
meta_693	4-Androsten-11β-ol-3,17-dione 2	0.451227	0.000564	1.868318	down
meta_697	Analyte 992	0.295608	0.003646	1.814251	down

meta_700	Tetrahydrocorticosterone 2	0.469944	0.005299	1.718901	down
meta_703	Analyte 999	0.478201	0.018176	1.521879	down

Note: “up” indicates the up-regulated DEMs in the comparison while “down” indicates down-regulated DEMs. VIP: variable importance in projection.

Table S7. Differentially expressed metabolites (DEMs) from the S2_vs_S5 comparison.

#ID	DEM name	Fold change	*P* value	VIP	Regulation
meta_54	Analyte 80	0.395492	0.000496	1.698705	down
meta_62	lactic acid	2.686551	0.023374	1.346093	up
meta_197	Isoleucine	2.035431	0.000787	1.650067	up
meta_248	Halostachine 1	0.210944	0.000109	1.794323	down
meta_262	Analyte 371	0.278055	0.00013	1.773462	down
meta_307	4-Acetamidobutyric acid 1	0.377085	0.000753	1.694503	down
meta_347	4-Hydroxyphenylethanol	0.231677	0.000658	1.701202	down
meta_364	Analyte 520	0.243683	3.96E-05	1.828751	down
meta_374	tartaric acid	0.381975	4.21E-05	1.76495	down
meta_395	Ribonic acid, γ-lactone	2.455181	0.011876	1.409019	up
meta_403	Synephrine 2	0.390264	0.006923	1.3661	down
meta_480	glucose 1	5.279526	1.4E-05	1.876897	up
meta_510	Analyte 720	0.390842	3.02E-05	1.808912	down
meta_573	pyridoxal phosphate 1	0.343795	0.001058	1.638424	down
meta_577	Analyte 809	0.349	5.37E-05	1.761312	down
meta_588	Analyte 825	0.445036	0.001571	1.552084	down
meta_591	Analyte 828	0.461567	0.000216	1.75747	down
meta_606	D-erythro-sphingosine 2	2.020828	0.00933	1.329825	up
meta_659	trehalose	0.475913	0.001292	1.66853	down
meta_671	unknown	2.394235	0.036246	1.225329	up
meta_684	Galactinol 2	2.945796	0.002193	1.581612	up
meta_693	4-Androsten-11β-ol-3,17-dione 2	0.411756	0.000128	1.744269	down
meta_697	Analyte 992	0.224974	0.002282	1.627957	down

meta_698	Analyte 993	0.448815	0.001834	1.645455	down
meta_700	Tetrahydrocorticosterone 2	0.427491	0.004146	1.575147	down
meta_703	Analyte 999	0.491207	0.02021	1.312458	down

Note: “up” indicates the up-regulated DEMs in the comparison while “down” indicates down-regulated DEMs. VIP: variable importance in projection.

Table S8. Differentially expressed metabolites (DEMs) from the S4_vs_S5 comparison.

#ID	DEM name	Fold change	*P* value	VIP	Regulation
meta_364	Analyte 520	0.462474	0.010013	1.72315	down
meta_538	Linoleic acid methyl ester	3.006574	0.018396	1.514995	up
meta_684	Galactinol 2	2.347046	0.004582	1.708342	up

Note: "up" indicates the up-regulated DEMs in the comparison while "down" indicates down-regulated DEMs. VIP: variable importance in projection.
